# Hepatoprotetive, Cardioprotective and Nephroprotective Actions of Essential Oil Extract of Artemisia sieberi in Alloxan Induced Diabetic Rats 

**Published:** 2012

**Authors:** Fawzi Irshaid, Kamal Mansi, Ahmad Bani-Khaled, Talal Aburjia

**Affiliations:** a *Department of Biological Sciences, Al al-Bayt University, P.O.BOX 130040, Al-Mafraq 25113, Jordan.*; b *Department of Pharmacy, University of Jordan, Amman 11942, Jordan. *

**Keywords:** *Artemisia sieberi*, Cholesterol, Essential oil, Insulin, Metformin, Uric acid

## Abstract

The aim of the current study is to evaluate the potential mechanism of antidiabetic action of the essential oil of *Artemisia sieberi *and its effects on some hematological and biochemical parameters in alloxan induced diabetic rats. Extraction of the essential oil from aerial parts of *A. sieberi *was preformed by hydrodistillation. Fifty rats were divided into five groups. Groups I and II normal rats given 1 mL/day of dimethyl sulfoxide and 80 mg/kg BW of this oil extract, respectively. Groups III, IV and V diabetic rats given 1 mL/day of dimethyl sulfoxide, oil extract (80 mg/kg BW) and metformin (14.2 mg/kg BW), respectively. Several hematological and biochemical parameters were assessed. Oral administration of the extract resulted in a significant reduction in the mean values of blood glucose, glucagon, cholesterol, triglyceride, LDL-C, ESR, urea, uric acid, creatinine accompanied by an increase in the mean values of the total protein, albumin, insulin, HDL-C, neutrophile count and PCV in diabetic rats. No significant changes in these parameters were found in the control group. The effects produced by this extract were closely similar to a standard antidiabetic drug, metformin. In conclusion, the present study indicates that the essential oil extract of *A*. *sieberi *appears to exhibit cardioprotective, nephroprotective and hepatoprotective activities in alloxan induced diabetic rats.

## Introduction

*Diabetes mellitus (DM) increases risk of several serious health problems or complications including hyperlipidemia, poor metabolic control, nephropathy, hepatopathy and cardiomyopathy (* 1 *-* 3 *). These complications are considered the leading causes for death among these patients. Thus, an early control of DM is recommended as one of the main strategies to prevent these complications and increase the life span of these patients. Several synthetic compounds have been used as therapeutic drugs for control of DM, including metformin (* 4 *-* 6 *). This drug is widely used in Jordan to regulate blood glucose level. However, most of synthetic drugs just regulate blood glucose level and does not completely cure DM and prevent or delay the onset of its complications (* 1 *-* 3 *). Therefore, the insufficient protection by these drugs necessitates the need for new treatment to prevent or delay these complications.*

*The use of natural products such as plant extracts is a common practice in Jordan for relieving and treating several diseases including DM (* 7 *-* 9 *). The most widely used plant species in treating DM in Jordan is *Artemisia sieberi *(*A. *sieberi*). This perennial shrub belongs to family Asteraceae and has a strong aromatic smell and a bitter taste. It grows abundantly in arid areas of Middle Eastern countries and other part of the world (10-12). This valuable plant can also be used to treat other ailments in the various traditional systems of medicine. Recently, we have demonstrated that essential oil extract derived from *A. sieberi *given as a repeated daily dose of 80 mg/Kg body weight (BW) for six weeks exhibits antidiabetic activity in a diabetic animal model produced by the injection of alloxan in rats (13). However, the mechanism of antidiabetic action of this essential oil has not yet been determined or investigated. Thus, we recommend more studies to be conducted to explore more about the role of this extract in the treatment and prevention of diabetes mellitus (DM) and its complications. Based on these data, the current investigation is amid to determine the potential mechanism of antidiabetic action of this extract. This investigation is also amid at exploring more about other potential biological and pharmacological activities of this extract, including cardioprotective, nephroprotective and hepatoprotective activities.

## Experimental

*Plant material and essential oil extraction*

*A. sieberi*, locally known as Shih, was collected during spring of 2009 just before flowering from Al-Mafraq area, approximately 68 Km North-East of Amman, the capital city of Jordan. The plant was identified and authenticated by Professor J. Lahham, taxonomist, at the herbarium of the Department of Biological Sciences, Faculty of Sciences, Yarmouk University, Irbid, Jordan. The voucher specimen (No.AHE-1-007) was deposited in the Department of Biological Sciences, Faculty of Sciences, Al al-Bayt University, Al-Mafraq, Jordan. Extraction of the essential oil from aerial parts of *A. sieberi *was preformed as described previously (13).

*Experimental animals and induction of diabetes in rats*

Wister rats (weighing 155-183 g) were purchased from the animal house of the Jordan University of Science and Technology, Irbid, Jordan, and were used for the present study. They were housed under standard laboratory conditions (12 h light/dark cycle, temperature of 25 oC and relative humidity of 45%) throughout the experimental periods. They had access to normal food (Top Fed, Sapele) and water *ad libitum. *The animals were daily checked and monitored for any sign of toxicity or changes during the entire period of experiment.

A single dose of 150 mg/Kg BW of alloxan monohydrate (*BOH Chemical***, ***LTD***, **Poole, *England*) was used to induce diabetes in experimental rats. Induction of diabetes was conducted as described in the previous experiment (13). After two weeks, rats with blood glucose of 200 mg/dL or more were classified as diabetic rats and were used for the subsequent experiments.

*Experimental design*

Fifty rats were randomly divided into five experimental groups of ten rats each. Groups I and II consisted of normal rats. Group I received only dimethyl sulfoxide DMSO (0.5 ml/kg BW) and served as control group. Group II was given 80 mg/kg BW of *A. sieberi *essential oil extract. Groups III, IV and V consisted of alloxan-induced diabetic rats. Group III received only DMSO (0.5 ml/kg BW). Group IV was given *A. sieberi *essential oil extract (80 mg/kg BW) and Group V was given metformin (14.2 mg/kg BW). Metformin was purchased from Bristol-Myers Squibb Company, UK. The essential oil extract and metformin were daily given, using an intragastric tube for 6 weeks. All rats were maintained in these treatment regimens for six weeks with free access to food and water. At the end of the experimental period, blood samples were taken from these experimental rats by cardiac puncture protocol. Rats were sacrificed by cervical dislocation under light ether anesthesia. These experiments complied with the guidelines of our animal ethics committee, which was established in accordance with the internationally accepted principles for laboratory animal use and care.

*Hematological analysis*

The cell blood count (CBC) is usually performed on an automated hematology analyzer using whole well-mixed blood to which EDTA is added to prevent clotting. ESR was determined using Westengren method. Differential leukocyte count was conducted on Geimsa stained blood smears. Blood glucose level was measured immediately by Haemo-Glukotest (20-800R) glucose strips supplied by M/S Boehringer Mannheim India Ltd.

*Plasma lipid profiles*

Lipid profile (total cholesterol (TC), triglycerides (TG), high-density lipoprotein cholesterol (HDL-C) and low-density lipoprotein cholesterol (LDL-C)) were measured by an enzymatic method using Bio-Merieux Kit (Bio- Meraux Lab reagent and product, France).

*Total protein, albumin, urea, uric acid and creatinine analyses*

Total protein, albumin, blood urea, uric acid and creatinine levels were also determined by using Bio-Merieux Kit (Bio- Meraux Lab reagent and product, France).

*Insulin and glucagon determination*

Serum insulin and glucagon were measured through radioimmunoassay methods (CEA-JRE-SORIN Firm, France).

*Statistical analysis*

The results were expressed as mean ± standard deviation. Differences between control and experimental groups were estimated using student›s t-test analysis. Within group comparisons were performed by analysis of variance using ANOVA test. Differences were considered significant if P-value was less than 0.05.

## Results and Discussion

To evaluate the effect of treatment of *A. sieberi *essential oil extract at 80 mg/Kg BW on some selected blood parameters, blood samples were taken weekly to monitor any change in blood glucose levels. The results for the effect of the essential oil extract of *A*. *sieberi *in the mean values of some hematological parameters on normal and diabetic rats are presented in Table 1. 

**Table 1:** Effect of essential oil of *Artemisia sieberi *on levels of some hematological parameters in normal and alloxan induced diabetic rats after six weeks of treatment

**Parameters**	**Groups**				
	**I**	**II**	**III**	**IV**	**V**
Blood glucose	95 ± 9.6	101 ± 11.5	369 ± 26.8*	204 ± 29.3 **	188 ± 19.5**
Hb (g/dL)	12.0 ± 1.3	12.3 ± 1.0	10.8 ± 1.0	11.4 ± 0.8	12.3 ± 1.3
RBCs x 106/ μL	5.8 ± 0.7	5.9 ± 0.9	4.6 ± 0.5	5.0 ± 0.6	5.2 ± 0.9
WBCs x 103/ μ	7.4 ± 1.0	6.5 ± 0.8	4.0 ± 1.4	3.9 ± 0.8	3.8 ± 0.7
Neutrophils%	32.8 ± 3.7	36.9 ± 4.0	24.6 ± 3.2*	36.7 ± 3.4**	39.7 ± 3.6**
Basophiles%	2.7 ± 0.2	2.6 ± 0.9	3.5 ± 0.7	3.9 ± 0.3	3.5 ± 0.4
Eosinophils %	3.5 ± 0.2	4.7 ± 0.6	4.4 ± 0.6	4.9 ± 0.4	4.5 ± 0.41
Lymphocytes%	63.7 ± 3.6	64.4 ± 4.2	63.5 ± 4.2	58.8 ± 3.7	60.3 ± 3.8
PCV	37.7 ± 2.7	38.5 ± 1.9	30.7 ± 2.3*	35.6 ± 2.7**	35.4 ± 2.6**
ESR	14.6 ± 2.4	12.6 ± 1.8	20.6 ± 2.4*	16.5 ± 1.6**	14.4 ± 2.9**

Values (mg/dL) are the mean values ± standard deviation of 10 rats; *: Statistically significant when compared to control group (I) at p < 0.05; **: Statistically significant when compared to untreated diabetic group (III) at p < 0.05

Our data indicated that there were significant decreases (p < 0.05) in the mean values of blood glucose levels in alloxan-diabetic rats treated with *A*. *sieberi *essential oil extract (Group IV) when compared to the untreated diabetic rats (Group III) during the entire period of the study. It also showed that there were significant decrease (p < 0.05) in the mean values of neutrophile count and PCV accompanied by a significant increase (p < 0.05) in the mean values of ESR in the untreated diabetic rats compared to the respective control rats (Group I). While an oral injection of 80 mg/Kg BW of *A. sieberi *essential oil extract in diabetic rats (Groups IV) caused a significant increase (p < 0.05) in the mean values of neutrophile count and PCV accompanied by a significant reduction (p < 0.05) in the mean values of ESR, no significant alterations or fluctuations were found in the mean values of other blood parameters including hemoglobin, RBC, basophile, eosinophile and lymphocyte counts in the untreated diabetic rats relative to the control rats. The effect of this essential oil dose was comparable to that of the metformin-administered rats.

The average value of insulin was significantly lower (p < 0.05) in the untreated diabetic rats compared to the respective control rats, as shown in Table 2. On other hand, there were significant increases (p < 0.05) in the mean value of insulin in diabetic group treated with essential oil of *A. *

**Table 2:** Effect of essential oil of *Artemisia sieberi *on insulin and glucagon levels in normal and alloxan induced diabetic rats after six weeks of treatment

**Parameters**	**Groups**					
	**I**	**II**	**III**	**IV**	**V**	
Insulin (mg/mL)	5.8 ± 0.3	6.1 ± 0.4	3.4 ± 0.1*	4.8 ± 0.1**		3.6 ± 0.2
Glucagon (pg/mol)	34.8 ± 4.6	38.7 ± 3.9	54.7 ± 8.8**	44.7 ± 3.7**		47.9 ± 6.8**

Values (mg/dL) are the mean values ± standard deviation of 10 rats; *: Statistically significant when compared to control group (I) at p < 0.05; **: Statistically significant when compared to untreated diabetic group (III) at p < 0.05.

*sieberi *as compared to the untreated diabetic rats during the entire period of the study. The insulin mean value in diabetic rats treated with metformin (Group V) was found to be closely similar to that in the untreated diabetic rats given only DMSO. Of note, the insulin mean value significantly reduced in the diabetic rats treated with metformin (Group V) relative to the control rats. Furthermore, the diabetic rats experienced significant increased (p < 0.05) in the mean value of glucagon when compared with the control rats; whereas the mean value of glucagon in the diabetic rats treated with *A. sieberi *essential oil extract (Group IV) or metformin was significantly (p < 0.05) lower than that of the untreated diabetic rats. The insulin mean value was not significantly altered in the normal rats treated with the essential oil extract (Group II) relative to the control rats.

As can be seen in Table 4, the mean values of urea, uric acid and creatinine were significantly higher in the untreated diabetes rats as compared to the control rats (p < 0.05). Treatment of the diabetic rats with *A. sieberi *essential oil extract and metformin for sex weeks (Groups IV and V) caused a significant decrease in urea, uric acid and creatinine as compared to the untreated diabetic group (p < 0.05). In addition, the average values of total protein and albumin levels in the untreated diabetic rats were significantly lower (p < 0.05) than that of the control rats. On other hand, in the diabetic groups treated with *A. sieberi *essential oil extract or metformin, the average values of total protein and albumin levels significantly (p < 0.05) increased, as compared with those of the untreated diabetic group.

Recently, our laboratory clearly demonstrated antihyperglycemic action of the essential oil derived from *A. sieberi *(13). Thus, an attempt has been made in the current work to investigate the potential mechanism of antidiabetic action of this extract in alloxan induced diabetic rats.

Our current study demonstrated that continuous oral administration of the essential oil extract of *A. sieberi *for six weeks significantly decreased blood glucose levels in the diabetic rats by 45% compared with the decreases of 49% with the metformin, a well known antidiabetic drug. Also our data revealed for the first time that this extract produced a significant increase in insulin level accompanied by significant reduction of glucagon and blood glucose levels in the diabetic rats. By contrast, in the normal rats, this extract had no effects on all of the investigated hematological and biochemical parameters during this study. The difference in these observations can be explained through the normal homeostasis mechanisms, such as that of the secretion of regulatory hormones. These hormones usually operate in the normal animals to maintain the normal homeostasis environment.

Furthermore, our data clearly indicated that the destruction of *β*-cells in our study appears to be partially, but not completely. This is because the plasma insulin level in the diabetic rats are about 58**% **of that in the normal rats. Therefore, our data suggest that antidiabetic action of *A. sieberi *essential oil might be associated with its ability to stimulate or increase insulin production from the pancreatic *β*-cells as well as inhibition of glucagon secretion from the pancreatic *β*-cells, since chronic administration of this extract has positive effect on the plasma insulin level and negative effect on the plasma glucagon level in diabetic rats. This is in agreement with previous studies (14, 15). These studies reported that there was selected destruction of pancreatic islet *β*-cells in alloxan induced diabetic model, since some *β*-cells do survive and plasma insulin levels in the diabetic rats are about 22% of that in normal rats. These studies suggest that the insulin secretion might be stimulated in the residual *β*-cells of these diabetic animals. Our data also revealed that metformin did not change the insulin plasma level. Moreover, according to previous reports, the hypoglycemic action of metformin in diabetic patients has been proposed via decreased glucose production, increased fatty acid oxidation in hepatocytes, and/or increased glucose uptake in skeletal muscle (4, 6, 16). Thus, our findings suggest that the mechanism of antidiabetic action of this essential oil extract appears to differ from those already described for metformin. Thus, the antidiabetic action of this extract might be mediated through distinct compounds. Moreover, we can not exclude the possibility that the antidiabetic action of this oil extract might be due to its ability to stimulate the proliferation of some of the survived pancreatic *β*-cells.

**Table 3:** Effect of essential oil of *Artemisia sieberi *on plasma lipid profiles in normal and alloxan induced diabetic rats after six weeks of treatment

**Parameters**	**Groups**				
	**I**	**II**	**III**	**IV**	**V**
Total Cholesterol	114 ± 8.3	108 ± 10.5	179 ± 21.45*	152 ± 18.8**	142 ± 16.7**
Triglyceride	75 ± 11.6	82 ± 9.3	128 ± 19.4*	97 ± 19.5**	95 ± 17.8 **
HDL-C	32 ± 5.6	31 ± 3.2	22 ± 4.3*	30 ± 2.6**	28 ± 3.4**
LDL-C	30 ± 4.7	27 ± 3.6	52 ± 11.5*	37 ± 9.4**	41 ± 11.8**

Values (mg/dL) are the mean values ± standard deviation of 10 rats; *: Statistically significant when compared to control group (I) at p < 0.05; **: Statistically significant when compared to untreated diabetic group (III) at p < 0.05.

In addition to marked hyperglycemia, our result revealed that the alloxan diabetic rats developed notable hyperlipidaemia. Diabetes induced hyperlipidaemia was observed in diabetic experimental animal models, and it is associated with the increase of mobilization of fat from fat cells and lipid metabolism due to the inability to utilize glucose properly (16-20). This is very important, since elevated concentrations of cholesterol, triglyceride and LDL-C are important risk factors in the development of artheriosclerosis in diabetes mellitus. For the first time our study revealed that this extract normalized serum lipids (cholesterol, triglyceride, HDL-C, LDL-C) closely to the level of the control or normal rats. Our findings are consistent with a recent study by Bavarva and Narasimhacharya (2010) which reported that leaves of *Leucas cephalotes *lowered both plasma and hepatic lipid profiles (total lipid, triglycerides and cholesterol) and LDL-C while elevating the HDL-C levels (18). They suggest that these improvements in lipid profiles are most likely due to its insulin-like actions of the leaf extract of *Leucas cephalotes*. Similarly, a previous study done by Lopes-Virella *et al. *(1983) also reported that diabetic patients taken insulin injections exhibited both high lipoprotein lipase activity and low level of plasma triglyceride concentrations (19). Thus, it can be concluded that the enhancement of insulin secretion or level is accompanied by enhancement of glucose utilization as well as a reduction of lipid level in diabetic rats. It is possible to suggest that the mechanism(s) of antihyperlipidemic effect of the A. *sieberi *might be similar to some of those suggested for anti-diabetic plants exhibiting antihyperlipidemic activity, such as activation of lipoprotein lipase, insulin-mediated lipolytic activity or inhibition of lipogenic enzymes or hormone-sensitive lipase (15-20).

Moreover, this study manifested a significant decrease in serum total protein and albumin levels in the untreated diabetic rats, whereas serum total protein and albumin levels significantly increased after the administration of this essential oil extract. Similar results were obtained when metformin was administered orally in the alloxan-induced diabetic rats. The serum total protein and albumin levels can also be used as an indicator of liver function. These results suggest that this extract can improve some biochemical parameters that are related to liver functions. Hyperglycemia has also been recently implicated in initiation and development of various types of diabetic complications. Nephropathy is one of these serious microvascular complications that has been observed in diabetic individuals (2). In addition, blood urea and creatinine concentrations were increased among uncontrolled diabetic individuals and this increase could be a result of impaired renal function due to an increased blood glucose level. Through this study it has been revealed, for the first time, that the mean values of these end products in the serum increased in untreated diabetic rats, while they significantly decreased after the administration of this essential oil extract. Thus, this oil extract might improve the renal function which, in turn, leads to reduction in these end products. It was reported that diabetic individuals had lower serum albumin concentrations as well as higher serum uric acid and urea levels than nondiabetic individuals (21, 22). Thus, the reduction in urea and creatinine levels can probably be explained through a reduction in blood glucose level.

DM is also considered as a risk factor for cardiovascular diseases, and the elevated serum uric acid has also been linked to these types of diseases, especially when accompanied with high triglyceride and low HDL. Moreover, high levels of serum uric acid, urea and creatinine may act as an indicator of kidney problems. Thus, it is possible to suggest that this essential oil extract might play an important role in reducing the risk of kidney problems as well as cardiovascular diseases via lowering serum urea, uric acid, creatinine as well as improving lipid profile. The beneficial effects that have been observed for the first time in our study are indications of the safety of the essential oil extract of *A. sieberi*, and hence it is worth trying to study the effects of this extract on some voluntary diabetic patients.

The study of the literature indicated that free radicals are one of the main contributors to development of DM as well as its complications (1, 18, 23-27). It is also worth mentioning that alloxan can induce rapid death of *β*-cells of pancreas, resulting in partial or complete loss of insulin production and leading to the development of hyperglycemia and its complications in experimental animals; and this action of alloxan was mediated by formation of free radicals (23). Moreover, a wide range of studies have strongly supported the notion that antioxidant compounds derived from plant extracts might play a vital role in the treatment of DM and prevent or delay its complications (1, 23-28). Thus, it possible to suggest that the above mentioned protective effects of *A*. *sieberi *could be due to the presence of high levels of antioxidant compounds in the essential oil extract of this plant. Direct support for this notion was also confirmed by identification and characterization the components of the essential oil derived from *Artemisia *species by our lab and others (12, 23, 27-29). According to these analyses, the essential oil is the mixture of a variety of lipid soluble and volatile compounds such as terpenes and terpenoids, phenol-derived alcohol, ketone and monotepenes compounds, and aliphatic components, and most of them were characterized as antioxidants.

**Table 4:** Effect of essential oil of *Artemisia sieberi *on total protein and albumin, urea, uric acid and creatinine levels in normal and alloxan induced diabetic rats after six weeks of treatment

**Parameters**	**Groups**				
	**I**	**II**	**III**	**IV**	**V**
Total protein (g/dL)	8.0 ± 1.0	7.7 ± 0.8	5.2 ± 0.7 *	6.4 ± 0.8**	7.4 ± 1.0 **
Albumin(g/dL)	3.4 ± 0.7	4.3 ± 0.9*	1.8 ± 0.5*	2.9 ± 0.2**	3.0 ± 0.2**
Urea (mg/dL)	27.8 ± 3.6	29.8 ± 2.5	37.4 ± 6.5*	31.6 ± 4.4**	29.7 ± 5.6**
Uric acid (mg/dL)	1.6 ± 0.4	1.8 ± 0.7	2.6 ± 0.8*	1.7 ± 0.2**	1.6 ± 0.2**
Creatinine (mg/dL)	1.0 ± 0.1	1.0 ± 0.2	2.7 ± 0.7*	1.4 ± 0.2**	1.3 ± 0.3**

Values (mg/dL) are the mean values ± standard deviation of 10 rats; *: Statistically significant when compared to control group (I) at p < 0.05; **: Statistically significant when compared to untreated diabetic group (III) at p < 0.05.

In conclusion, this is the first study to reveal that oral administration of the essential oil extract of *A*. *sieberi *exhibit cardioprotective, nephroprotective and hepatoprotective activities via enhances insulin production and decreases glucagon production in the alloxan-induced diabetic rats. Thus, oral use of this extract might positively affect the functional capacities of various rat tissues, particularly blood, heart, kidney and liver against toxic action of alloxan compound (dose of 150 mg/Kg BW). These findings clearly support the traditional use of this medicinal plant in treatment of diabetes mellitus and shed more light on the efficacy of this plant. Thus, *A*. *sieberi *appears to be a valuable plant and ideally suited to be used in treatment of DM and prevent or delay the onset of its complications in humans, since this is a non-toxic plant.
